# Three-dimensional coherent X-ray diffractive imaging of whole frozen-hydrated cells

**DOI:** 10.1107/S205225251501235X

**Published:** 2015-08-20

**Authors:** Jose A. Rodriguez, Rui Xu, Chien-Chun Chen, Zhifeng Huang, Huaidong Jiang, Allan L. Chen, Kevin S. Raines, Alan Pryor Jr, Daewoong Nam, Lutz Wiegart, Changyong Song, Anders Madsen, Yuriy Chushkin, Federico Zontone, Peter J. Bradley, Jianwei Miao

**Affiliations:** a Biological Chemistry, UCLA-DOE Institute for Genomics and Proteomics, University of California, Los Angeles, CA 90095, USA; b Department of Physics and Astronomy and California NanoSystems Institute, University of California, Los Angeles, CA 90095, USA; c Department of Physics, National Sun Yat-sen University, Kaohsiung 80424, Taiwan; d Carl ZEISS X-ray Microscopy Inc., Pleasanton, CA 94588, USA; e State Key Laboratory of Crystal Materials, Shandong University, Jinan 250100, People’s Republic of China; f Department of Microbiology, Immunology, and Molecular Genetics, University of California, Los Angeles, CA 90095, USA; g Department of Applied Physics, Stanford University, Stanford, CA 94305, USA; h Department of Physics, Pohang University of Science and Technology, Pohang 790-784, South Korea; i NSLS-II Photon Sciences Division, Brookhaven National Laboratory, Upton, NY 11973, USA; j European X-ray Free Electron Laser, Albert-Einstein-Ring 19, Hamburg 22761, Germany; k ESRF, The European Synchrotron, 71 Avenue des Martyrs, Grenoble, France

**Keywords:** coherent diffractive imaging, cryo-CDI, three-dimensional imaging, three-dimensional cellular structure, coherent diffraction, X-ray imaging, *Neospora caninum*

## Abstract

A structural understanding of whole cells in three dimensions at high spatial resolution remains a significant challenge and, in the case of X-rays, has been limited by radiation damage. By alleviating this limitation, cryogenic coherent diffractive imaging (cryo-CDI) can in principle be used to bridge the important resolution gap between optical and electron microscopy in bio-imaging. Here, the first experimental demonstration of cryo-CDI for quantitative three-dimensional imaging of whole frozen-hydrated cells using 8 keV X-rays is reported. As a proof of principle, a tilt series of 72 diffraction patterns was collected from a frozen-hydrated *Neospora caninum* cell and the three-dimensional mass density of the cell was reconstructed and quantified based on its natural contrast. This three-dimensional reconstruction reveals the surface and internal morphology of the cell, including its complex polarized sub-cellular structure. It is believed that this work represents an experimental milestone towards routine quantitative three-dimensional imaging of whole cells in their natural state with spatial resolutions in the tens of nanometres.

## Abstract 2

Since its first experimental demonstration in 1999, coherent diffractive imaging (CDI) has been applied to image a broad range of samples using advanced synchrotron radiation, X-ray free-electron lasers, high harmonic generation and electrons. Here, the first experimental demonstration of cryogenic CDI for quantitative three-dimensional imaging of whole frozen-hydrated cells is reported. As a proof of principle, the three-dimensional mass density of the sub-cellular organization of a *Neospora caninum* cell is determined based on its natural contrast.

## 1. Introduction   

Microscopy has transformed our understanding of biology and medicine. By using novel imaging technologies and labelling techniques, optical microscopy can routinely study dynamic processes in living cells (Stephens & Allan, 2003 ▸). The typical resolution for optical microscopy is around 200 nm, although better resolution can be achieved in certain cases by using super-resolution fluorescence microscopes (Huang *et al.*, 2009 ▸). To achieve considerably higher resolution, electron microscopy is the method of choice. Electron tomography is currently the highest resolution imaging technique available to study non-identical structures in three dimensions (Scott *et al.*, 2012 ▸; Chen *et al.*, 2013 ▸; Lučić *et al.*, 2005 ▸). For radiation-hard inorganic materials, atomic-scale three-dimensional resolution has recently been achieved (Scott *et al.*, 2012 ▸; Chen *et al.*, 2013 ▸). For biological specimens such as pleomorphic macromolecular assemblies, viruses, organelles and cells, the resolution is currently limited to 3–5 nm by radiation damage to the specimens (Lučić *et al.*, 2005 ▸). However, the main constraint of electron microscopy is its limited applicability to thin or sectioned samples (typically ≤ 0.5 µm) due to the relatively small penetration depth of electrons (Scott *et al.*, 2012 ▸; Chen *et al.*, 2013 ▸; Lučić *et al.*, 2005 ▸). There is thus an important gap between optical and electron microscopy in terms of spatial resolution, sample thickness, labelling requirement, contrast mechanism and quantitative capability. Coherent diffractive imaging (CDI) is a rapidly growing imaging modality to bridge this gap for the following reasons (Miao *et al.*, 2015 ▸). First, due to the large penetration depth of X-rays, CDI can be used to image whole biological cells and biomaterials without the requirement of sectioning (Miao *et al.*, 2003 ▸; Shapiro *et al.*, 2005 ▸; Jiang *et al.*, 2008 ▸, 2010 ▸; Song *et al.*, 2008 ▸; Nishino *et al.*, 2009 ▸; Huang *et al.*, 2009 ▸; Lima *et al.*, 2009 ▸; de la Cuesta *et al.*, 2009 ▸; Nelson *et al.*, 2010 ▸; Giewekemeyer *et al.*, 2010 ▸; Nam *et al.*, 2013 ▸; Kimura *et al.*, 2014 ▸; Gallagher-Jones *et al.*, 2014 ▸; Bergh *et al.*, 2008 ▸; Seibert *et al.*, 2011 ▸; Schlichting & Miao, 2012 ▸). Second, compared with super-resolution fluorescence microscopy (Huang *et al.*, 2009 ▸), which allows for the selection of the fluorescently labelled molecules or molecular assemblies, CDI is based on the intrinsic mass density variations of biological specimens and accordingly enables quantitative three-dimensional imaging of the entire contents of cells, cellular organelles and biomaterials in their natural contrast (Miao *et al.*, 2003 ▸; Shapiro *et al.*, 2005 ▸; Jiang *et al.*, 2008 ▸, 2010 ▸; Song *et al.*, 2008 ▸; Nishino *et al.*, 2009 ▸; Huang *et al.*, 2009 ▸; Lima *et al.*, 2009 ▸; de la Cuesta *et al.*, 2009 ▸; Nelson *et al.*, 2010 ▸; Giewekemeyer *et al.*, 2010 ▸; Nam *et al.*, 2013 ▸; Kimura *et al.*, 2014 ▸; Gallagher-Jones *et al.*, 2014 ▸; Bergh *et al.*, 2008 ▸; Seibert *et al.*, 2011 ▸; Schlichting & Miao, 2012 ▸). Third, compared with zone-plate X-ray microscopy (Sakdinawat & Attwood, 2010 ▸; Weiß *et al.*, 2000 ▸; Le Gros *et al.*, 2005 ▸; Schneider *et al.*, 2010 ▸; Meirer *et al.*, 2011 ▸), CDI avoids the use of X-ray lenses and its resolution is only limited by the radiation damage imparted on biological specimens.

Since its first experimental demonstration in 1999 (Miao *et al.*, 1999 ▸), CDI has been employed to image a variety of biological specimens including biomaterials, whole cells, cellular organelles and viruses, utilizing synchrotron radiation (Miao *et al.*, 2003 ▸; Shapiro *et al.*, 2005 ▸; Jiang *et al.*, 2008 ▸, 2010 ▸; Song *et al.*, 2008 ▸; Nishino *et al.*, 2009 ▸; Huang *et al.*, 2009 ▸; Lima *et al.*, 2009 ▸; de la Cuesta *et al.*, 2009 ▸; Nelson *et al.*, 2010 ▸; Giewekemeyer *et al.*, 2010 ▸; Nam *et al.*, 2013 ▸) and X-ray free-electron lasers (Kimura *et al.*, 2014 ▸; Gallagher-Jones *et al.*, 2014 ▸; Bergh *et al.*, 2008 ▸; Seibert *et al.*, 2011 ▸; Schlichting & Miao, 2012 ▸). However, radiation damage has limited the applicability of CDI for high-resolution three-dimensional imaging of biological samples (Howells *et al.*, 2009 ▸; Shen *et al.*, 2004 ▸). One solution to alleviate the radiation damage problem is to keep the samples at cryogenic (liquid nitrogen) temperatures. Previous studies have suggested that cryo-CDI may be applied to image frozen-hydrated cells with a three-dimensional resolution of ∼10 nm (Howells *et al.*, 2009 ▸; Shen *et al.*, 2004 ▸). Although cryo-CDI has been demonstrated in two dimensions (Huang *et al.*, 2009 ▸; Lima *et al.*, 2009 ▸), it has thus far defied any experimental attempts to be achieved in three dimensions. Here, we report the first three-dimensional cryo-CDI of a whole frozen-hydrated cell using 8 keV X-rays. We chose to image the protozoan parasite *Neospora caninum*, which infects a wide variety of mammals and causes abortion in cattle and neuromuscular disease in dogs (Sohn *et al.*, 2011 ▸). *N. caninum* is a relative of the human pathogen *Toxoplasma gondii*, which causes disease in immune-compromised patients and neonates, and also a relative of *Plasmodium falciparum*, the causative agent of malaria (Sohn *et al.*, 2011 ▸; Baum *et al.*, 2008 ▸). A typical *N. caninum* cell has a banana-shaped body approximately 1–3 µm wide and 4–6 µm long. Like other apicomplexans, *N. caninum* undergoes several stages of development during its normal lifecycle. For the purpose of this study, we targeted imaging of the tachyzoite, a relevant stage in the parasite lifecycle during which it replicates quickly and infects most cell types within its host (Sohn *et al.*, 2011 ▸; Carruthers & Suzuki, 2007 ▸). In spite of its small size, the *N. caninum* parasite hosts a remarkable array of sub-cellular compartments including rhoptries, micronemes, dense granules, a conoid, apicoplast, nucleus, mitochondrion, ER and Golgi (Speer *et al.*, 1999 ▸). The sub-cellular structure of *N. caninum* tachyzoites is critical to their infectious nature and their survival both outside and within host cells (Sohn *et al.*, 2011 ▸). Using three-dimensional cryo-CDI, we imaged the surface and internal morphology of a *N. caninum* tachyzoite, including its complex polarized sub-cellular structure at ∼74–99 nm resolution. With further development, cryo-CDI can in principle be used to image whole frozen-hydrated cells with ∼10 nm resolution (Howells *et al.*, 2009 ▸; Shen *et al.*, 2004 ▸). As a quantitative three-dimensional imaging method (Song *et al.*, 2008 ▸; Jiang *et al.*, 2010 ▸), coupled with labelling and correlative approaches (Nelson *et al.*, 2010 ▸), cryo-CDI could facilitate the dissection of cellular and molecular pathways important to the movement, growth and disease states of a broad variety of cells including parasitic pathogens, whose inner workings are difficult to probe by other means.

## 2. Experimental methods   

### 2.1. Preparation of *N. caninum* tachyzoites   

The NC1 strain *N. caninum* was grown and maintained by serial passage in confluent monolayers of human foreskin fibroblasts grown in DMEM supplemented with 10% fetal calf serum plus penicillin, streptomycin and glutamine (Sohn *et al.*, 2011 ▸). A disperse suspension of *N. caninum* was prepared by extrusion from fibroblasts *via* passage through a syringe. A single cell suspension containing phosphate buffered saline, with 10% glycerol and 7% trehalose as cryo-protectants, was deposited on a 50 nm-thick silicon nitride membrane (Silson Ltd), custom mounted on a brass pin support. Only those membranes with isolated cells, which appeared visibly uncompromised and measured roughly 4–5 µm in their longest dimension, were used for our imaging experiments. During our experiment, we found that using a 50 nm-thick silicon nitride membrane as a sample holder can produce significantly better quality diffraction patterns than using a nylon or Kapton loop (Lima *et al.*, 2009 ▸).

### 2.2. Cryo preservation of *N. caninum* cells   

**Figure 1:**
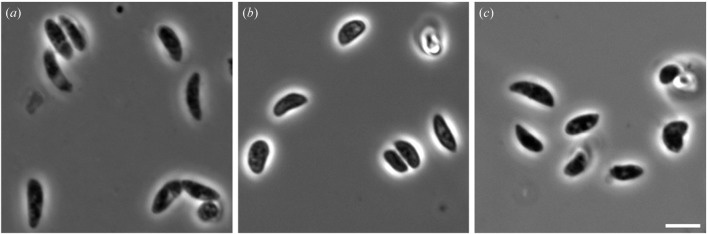
Examination of the cell morphology of rapidly frozen *N. caninum* with cryo-protectants. Extracellular *N. caninum* tachyzoites were resuspended in a tissue culture medium (*a*), cryo-protectants only (*b*), and cryo-protectants followed by rapid freezing in liquid nitrogen (*c*). The cells were imaged with an AxioCam MRm CCD camera and AxioVision software on an Axio Imager Z1 microscope (Zeiss) using a 100× oil immersion objective. Rapidly frozen tachyzoites with cryo-protectants exhibited similar morphology to those parasites that were resuspended in cryo-protectants only and in a tissue culture medium. Scale bar: 5 µm.

Preparation of frozen-hydrated specimens requires rapid freezing of a thin aqueous layer to create vitreous ice. This is conventionally achieved by rapid ‘plunge-freezing’ into liquid ethane. This method has been widely used in cryo-electron microscopy (cryo-EM) (Dubochet *et al.*, 1998 ▸). Our attempts at plunge-freezing of cells into liquid ethane and then transferring them into a nitrogen gas cryostream showed variable results as the sample-transferring step had a high probability of causing ice contamination. Ultimately, we found that high-quality single-cell diffraction was most reliably obtained when rapidly freezing cells directly into the nitrogen gas cryostream. This method required optimization of the cryo-protectant cocktail, as described in the previous section. The cryo-protectants we chose have been shown to preserve the integrity of cells during freezing in liquid nitrogen and to stave off the nucleation and growth of ice crystals (Pellerin-Mendes *et al.*, 1997 ▸; Rowe *et al.*, 1968 ▸). We further confirmed our protocol by comparing the morphology of *N. caninum* tachyzoites that were resuspended in a tissue culture medium, cryo-protectants only, and cryo-protectants followed by rapidly freezing in liquid nitrogen. Our phase contrast microscopy images show that rapidly frozen tachyzoites with cryo-protectants exhibited similar morphology to unfrozen parasites that were resuspended in a tissue culture medium and in cryo-protectants only (Fig. 1 ▸). However, there are drawbacks to the use of cryo-protectant agents, as these may thicken the vitreous ice layer and reduce contrast. Our future efforts are focused on uniform (high-pressure) freezing of samples, combined with focused ion beam milling (Lučić *et al.*, 2005 ▸), on substrates compatible with our instrument.

### 2.3. Experimental layout of a three-dimensional cryo-CDI microscope   

**Figure 2:**
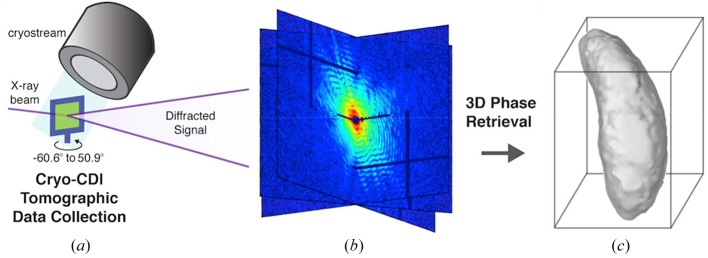
Schematic layout of a three-dimensional cryo-CDI microscope. (*a*) Diagram showing a silicon nitride membrane containing frozen-hydrated cells on a single-tilt piezo-electric stage, bathed in a nitrogen gas cryostream. A coherent X-ray beam impinges on a cell, from which a representative tilt series of 72 diffraction patterns was measured using a MAXIPIX detector (*b*). A three-dimensional diffraction pattern was assembled from the 72 two-dimensional diffraction patterns, from which the three-dimensional structure of the cell (*c*) was iteratively reconstructed by using the oversampling smoothness (OSS) algorithm (Rodriguez *et al.*, 2013 ▸).

The CDI experiments were conducted at the refurbished ID10 beamline at the European Synchrotron Radiation Facility (ESRF) as summarized in Table 1 ▸. An X-ray beam with *E* = 8 keV was selected from the undulator radiation by a pseudo channel-cut Si(111) monochromator (Δ*E*/*E* = 1.4 × 10^−4^). High-order harmonics were suppressed by a pair of Si mirrors reflecting at the grazing-incidence angle of 0.2°. A pair of roller blade slits closed to ∼7 µm × 7 µm was placed 0.5 m upstream from the sample and produced coherent illumination at the sample. Diffraction from the beam-defining slits was cleaned by a second pair of roller blade slits and finally by a Si corner placed in front of the sample. The combination of two pairs of slits and a Si corner allowed us to create a small and clean X-ray beam and significantly reduce the parasitic scattering from the upstream optics. The X-ray beam impinged on a silicon nitride membrane containing frozen-hydrated *N. caninum* cells, as shown in Fig. 2 ▸. X-ray diffraction patterns were recorded using a MAXIPIX 2 × 2 detector (Ponchut *et al.*, 2011 ▸), with a sample-to-detector distance of 5.29 m to meet the oversampling requirement (Miao *et al.*, 1998 ▸). The pixel size, detection area and image size of the MAXIPIX are 55 µm × 55 µm, 28.4 mm × 28.4 mm and 516 × 516 pixels, respectively.

### 2.4. Three-dimensional data acquisition of frozen-hydrated cells   

Rapidly frozen *N. caninum* tachyzoites with cryo-protectants were supported on an X-ray transparent silicon nitride membrane and bathed in a 100 K nitrogen gas stream to preserve the specimen during data acquisition (Fig. 2 ▸). Phase contrast optical microscopy images were used to confirm that rapidly frozen tachyzoites with cryo-protectants exhibited similar morphology to unfrozen parasites that were resuspended in culture medium (Fig. 1 ▸). The silicon nitride membrane was mounted onto a motorized stage with accurate three-dimensional translation and one-dimensional rotation capabilities, and scanned visually using an in-line optical microscope. Optical microscope images were correlated with diffraction patterns from the samples at all points scanned, in a mesh-grid pattern. Single isolated cells on the membrane were chosen for imaging on the basis of their preliminary diffraction and visual inspection.

Once a cell was chosen, fine alignment was performed by diffraction, *i.e.* positioning the beam at the point in which the diffraction pattern was strongest from the sample. For this reason, it is important to prepare well isolated cells with minimal background signal. Alignment by diffraction was performed at each tilt angle, with an approximate exposure time of 5 s per angle. Using this data acquisition system, we have collected several tilt series of diffraction patterns from frozen-hydrated cells with ∼5 h per tilt series. Figure S1 of the supporting information shows a representative tilt series of 72 diffraction patterns spanning approximately 111° (−60.6° to 50.9°). The tilt series was acquired based on the equal slope tomography (EST) scheme (Miao *et al.*, 2005 ▸). 37 positive and 34 negative angle patterns were collected each with an exposure time of 100 s. Data collection at high tilt angles was only limited by the geometry of the membrane support and the cryogenic cooling system. The total radiation dose imparted onto the frozen-hydrated cell was estimated to be 4.55 × 10^8^ Gy. A 0° pattern was collected multiple times during acquisition of the tomographic tilt series to monitor the structural integrity of the cell. A comparison of two independent 0° projections shows no appreciable structural change (Fig. S2 of the supporting information). A rise in background intensity towards the end of our tomographic acquisition scheme reveals potentially parasitic scattering from contamination or ice buildup near the sample (Fig. S1 of the supporting information). Otherwise, pattern features were generally conserved during data acquisition and the overall intensity in the patterns did not show appreciable decay.

## 3. Three-dimensional image reconstruction and post data analysis   

### 3.1. Post data processing and assembly of a three-dimensional diffraction pattern   

**Figure 3:**
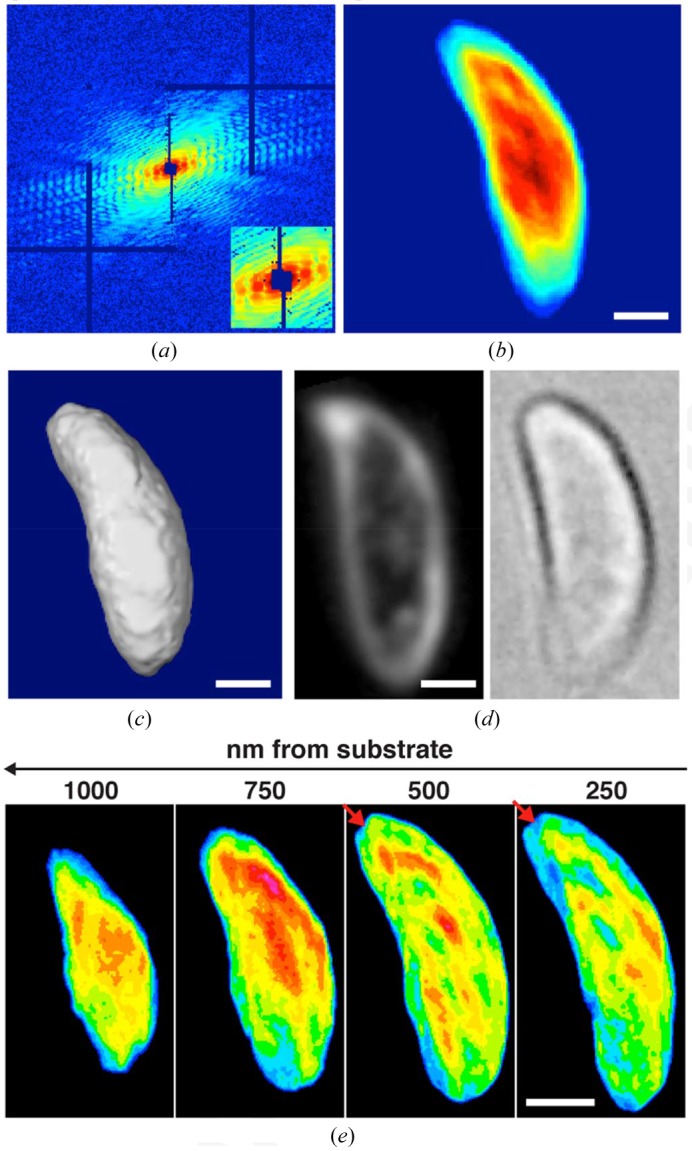
Three-dimensional structure determination of a whole frozen-hydrated *N. caninum* cell. (*a*) A representative diffraction pattern taken at the 0° tilt angle in which the horizontal and vertical bars are caused by the tiling of the 2 × 2 modules of the MAXIPIX detector (Ponchut *et al.*, 2011 ▸). The inset shows an enlarged version of the low-spatial-frequency region of the pattern where the missing data at the centre are due to a beam stop. A two-dimensional projection (*b*) and an isosurface rendering (*c*) of the reconstructed three-dimensional cell at the 0° tilt angle. The colour scale for the projection represents electron density. (*d*) Dark-field and bright-field optical microscope images of similar cells are shown enlarged to an equivalent scale for comparison. (*e*) A series of thin slices through the cell at a distance ranging from 250 to 1000 nm away from the silicon nitride substrate, in which the red arrows indicate a conoid-like region. Images in (*a*), (*b*), (*e*) are false coloured: red, yellow, green, blue and black range from high, medium and low to no electron density. Scale bars: 500 nm.

To remove the background scattering, two diffraction patterns were acquired for every tilt angle by moving the cell in and out of the X-ray beam; again stressing the importance of well isolated cells with a clean surrounding background. The two diffraction patterns were scaled on a per-pixel basis, accounting for non-linearity and flat-field correction to the detector (Ponchut *et al.*, 2011 ▸). The background pattern was then subtracted from the diffraction pattern of the cell to obtain corrected intensities. A portion of the low-resolution region of the resulting diffraction pattern, including the direct beam and the first speckle were blocked by a beam stop [Fig. 3(*a*) ▸, and Fig. S3 of the supporting information]. Some of these pixels were recovered based on centro-symmetry, and all other pixels assigned the average of their symmetric counterparts. Pixels with missing values were considered unknown. All 72 projections were processed in this way using a data analysis and quality control pipeline implemented in Matlab (Xu *et al.*, 2011 ▸). By combining the post-processed two-dimensional diffraction patterns, a three-dimensional matrix of diffraction intensities was assembled. The assignment of intensities within this matrix was achieved *via* an interpolation procedure using an inverse distance-weighting scheme.

### 3.2. Three-dimensional phase retrieval   

Phase retrieval of the assembled three-dimensional diffraction pattern was achieved using the oversampling smoothness (OSS) algorithm (Rodriguez *et al.*, 2013 ▸). OSS iterates back and forth between real and reciprocal space. The OSS algorithm exploits the correlation information among voxels in the region outside of the defined support in real space by applying a series of frequency filters in reciprocal space. We first ran 100 independent reconstructions using the OSS algorithm, each of which used a cubic support and a random phase set as the initial input. Each reconstruction was monitored by *R*_rec_,
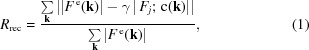
where 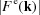 is the experimental Fourier modulus (*i.e.* square root of the diffraction intensities), 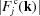 is the Fourier modulus calculated from a three-dimensional reconstruction in the *j*th iteration, and γ is a scaling factor. After 1000 iterations, we obtained 100 independent three-dimensional reconstructions, from which we selected the ten best with the lowest *R*_rec_. By averaging these ten reconstructions, we determined a tight three-dimensional support, which is slightly larger than the cell boundary. Using this tight support, we ran another 100 independent reconstructions, each with 1000 iterations. The final three-dimensional structure was the average of the best ten reconstructions with the smallest *R*_rec_ (approximately 25%). Based on the retrieved intensity of the central speckle for the final three-dimensional structure (Fig. S3), an estimate of the mass density was obtained, producing a quantitative three-dimensional structure of the cell and its features. Our phase retrieval process requires minimal manual intervention and the 100 independent reconstructions can be implemented through parallel computing. The computation time of running a 601 × 601 × 601 voxel array for 1000 iterations is ∼8 h on a Dual Intel Xeon CPU E5-2670 2.60 GHz computer system.

### 3.3. Estimation of three-dimensional resolution achieved   

**Figure 4:**
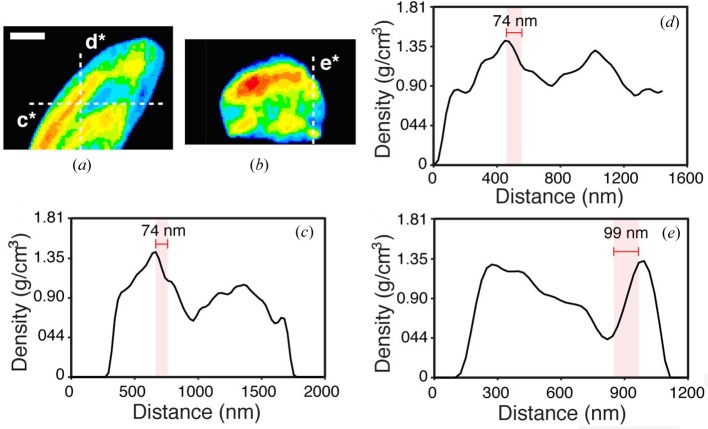
Resolution estimation for the three-dimensional cryo-CDI reconstruction. (*a*), (*b*) Two thin slices through the three-dimensional reconstruction of the cell. Dotted lines c*, d* and e* correspond to line scans along the *X*, *Y* and *Z* axes, respectively, where the X-ray beam is in the *Z* axis. Images are false coloured; red, yellow, green, blue and dark blue/black range from high, medium and low to no signal/density, respectively. Scale bar: 500 nm. (*c*)–(*e*) Line scans of c*, d* and e* indicate that a resolution of ∼74 nm, ∼74 nm and ∼99 nm was achieved in the three-dimensional reconstruction along the *X*, *Y* and *Z* axes, respectively. The lower resolution along the *Z* axis is due to the missing data (wedge) in that direction.

We quantified the resolution of our three-dimensional reconstruction using two independent approaches. First, we calculated the phase retrieval transfer function (PRTF) of our three-dimensional reconstructions (Chapman *et al.*, 2006 ▸) (Fig. S4 of the supporting information). Based on the PRTF = 0.5 criterion, a resolution of approximately 68 nm was estimated. Second, we performed line scans along the three primary axes of the three-dimensional reconstruction. A feature near the apical end of the cell provided strong contrast for estimating resolution. In the *X* and *Y* axes, a resolution of ∼74 nm was measured (Fig. 4 ▸). Along the *Z* axis (*i.e.* the direction of the X-ray beam), which suffered from missing data, our resolution estimation was ∼99 nm (Fig. 4 ▸). Collectively, these measurements indicate that a full-period resolution of ∼74–99 nm was achieved in our three-dimensional reconstruction.

### 3.4. Quantification of the radiation dose   

To quantify the radiation dose to the sample, we measured the coherent X-ray flux per unit area at the sample position to be 4.99 × 10^7^ photons µm^−2^ s^−1^. For a 100 s exposure, this corresponds to an intensity of *P*_t_/*A* = 4.99 × 10^9^ photons µm^−2^. The dose per projection, *D*_p_, was estimated as
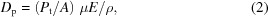
where μ is the linear absorption coefficient, ρ the density of the cell, and μ/ρ was estimated to be 9.9 cm^2^ g^−1^ with *E* = 8 keV. This gives a dose per projection *D*_p_ of 6.32 × 10^6^ Gy. The total dose (*D*_t_) imparted onto the frozen-hydrated cell was estimated to be 4.55 × 10^8^ Gy. With this dose, no noticeable deterioration was observed in the diffraction patterns (Fig. S2), which is consistent with previous CDI studies of frozen-hydrated cells (Huang *et al.*, 2009 ▸; Lima *et al.*, 2009 ▸; Howells *et al.*, 2009 ▸; Shen *et al.*, 2004 ▸).

### 3.5. Quantification of the mass density   

We first estimated the electron density of the final three-dimensional reconstruction of the *N. caninum* cell. To achieve this, we considered (Jiang *et al.*, 2010 ▸)
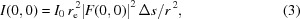
where 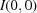 represents the number of diffracted photons at the central pixel of the MAXIPIX detector, 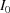 is the incident flux per unit area on the sample, 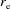 is the classical electron radius, 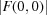 the total number of electrons inside the cell, 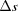 the area covered by this central pixel, and *r* the distance from the sample to the detector. In our experiment, 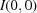 was determined to be 1.88 × 10^7^ photons (Fig. S3) and 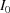 was measured to be 4.99 × 10^9^ photons µm^−2^, in which the sample was assumed to be uniformly illuminated by the incident X-ray beam. By substituting the numbers into equation (3), we calculated the total number of electrons inside the cell to be 2.08 × 10^12^. If we assume the cell to have a composition similar to that of a protein with empirical composition H_50_C_30_N_9_O_10_S_1_ and to be composed primarily of light elements (Giewekemeyer *et al.*, 2010 ▸), the relationship between molecular weight and the total number of electrons 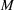 = 1.88*N*_e_ can be used to convert electron to mass density (Jiang *et al.*, 2010 ▸). As the volume of our reconstructed tachyzoite cell is 5.7 µm^3^, we calculated the average mass density of the cell to be 1.15 g cm^−3^.

### 3.6. Three-dimensional model building and rendering   

The final three-dimensional reconstruction was processed to obtain a three-dimensional model representing the cell bounds and the regions within the cell that may pertain to specific sub-cellular structures or organelles. The density per voxel of the three-dimensional reconstruction was used as a metric for demarcating regions. The IMOD suite was employed for this and subsequent model building tasks (Kremer *et al.*, 1996 ▸). The program includes the *3dmod* package, and facilitates semi-automatic model building, display and image processing on a per-slice basis, to build a three-dimensional model from the three-dimensional cell reconstruction (Kremer *et al.*, 1996 ▸). We relied on several manually chosen thresholds for semi-automatic segmentation. The cell bound was chosen using a threshold that excluded all voxels with density less than approximately 10% of that of the maximum density in the reconstruction. For regions within the cell, local contrast was used to outline objects of interest and their bounds. For each object, sections in between the manually outlined closed contours were interpolated automatically using *3dmod*. The final images were rendered using the image-processing programs *ImageJ*, *Amira* and *Chimera*, and assembled into figures using Adobe Illustrator.

## 4. Results and discussion   

### 4.1. Three-dimensional cryo-CDI reveals a *N. caninum* tachyzoite with a polarized sub-cellular structure   

The reconstructed three-dimensional density shows a banana-shaped cell with an overall size of ∼1.5 × 2 × 4 µm (Fig. 3 ▸). Projections of the three-dimensional density of the cell along its primary axes reveal characteristic views of a *N. caninum* tachyzoite. Figs. 3(*b*) and 3(*c*) ▸ show a two-dimensional projection and an isosurface rendering of the reconstructed three-dimensional cell at the 0° tilt angle, respectively. These projection images resemble but exhibit higher resolution than dark-field and bright-field optical microscope images of tachyzoites under similar conditions (Fig. 3*d* ▸). Fig. 3(*e*) ▸ shows a series of thin slices through the cell at a distance ranging from 250 to 1000 nm from the silicon nitride substrate, in which the red arrows indicate a conoid-like region at the apical end of the cell.

**Figure 5:**
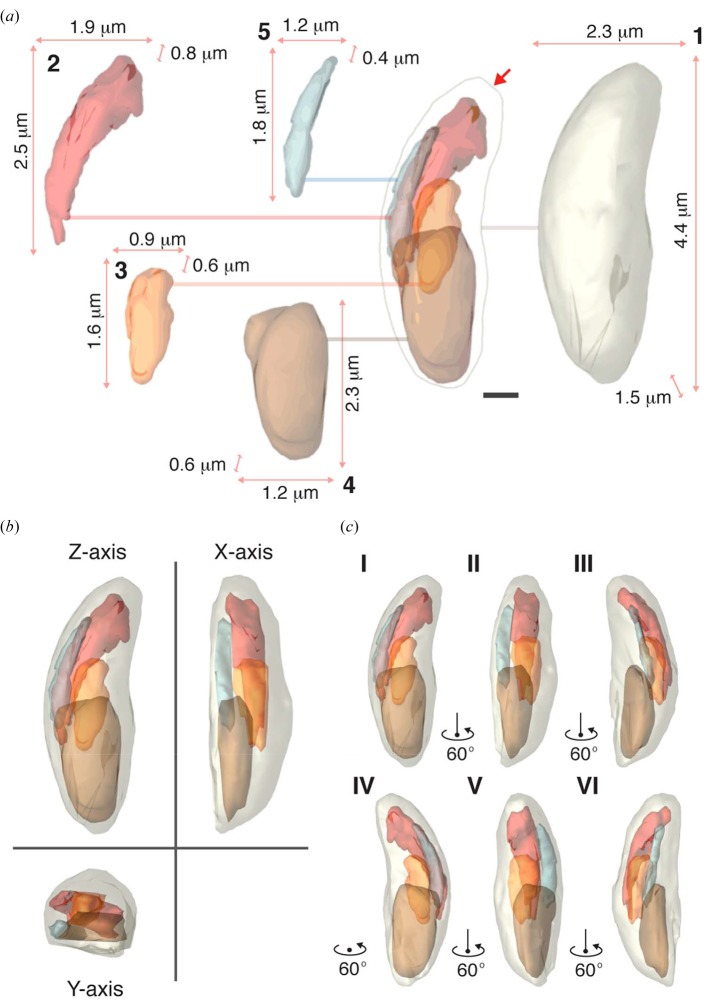
Three-dimensional sub-cellular structure of the frozen-hydrated *N. caninum* cell. (*a*) A deconstructed isosurface model of the *N. caninum* tachyzoite is shown with two-dimensional projections along the beam direction of three-dimensional renderings of each region chosen to be highlighted (see Table S1). At the centre, the boundary of the cell is shown, as well as each of the regions in its corresponding location within the cell. Regions are numbered and coloured according to their listing in Table S1. A line extends from each of these regions to their cut-outs, which are numbered and bound by the three lengths of the box that circumscribes them; scale bars: 500 nm. (*b*) Views of the three-dimensional isosurface model of the tachyzoite along the three primary axes. The *Z* axis corresponds to a view along the beam direction at the 0° tilt angle. (*c*) Starting from the 0° tilt angle, the *Z*-axis view in (*b*) is rotated about a primary axis, each frame (I–VI) in the montage is a 60° tilt apart from the previous.

Definitive identification of cellular organelles was difficult from the recovered three-dimensional density alone due in part to its limited resolution and the difficulty of segmenting nearly continuous features within its sub-cellular structure. However, we constructed a three-dimensional model from the tachyzoite density by outlining general features and boundaries for large sub-cellular structures, primarily focusing on the apical end of the cell (Fig. 5 ▸). Using semi-automatic segmentation, we distinguished between adjacent features based on their reconstructed intensities, the size and appearance of the segmented objects. At the apical end of the cell, a bud-like structure was recognizable as the apical tip which has a tapered structure from which the conoid is expected to emerge (Fig. 5 ▸). Underlying the apical tip, a network of dense tubulo­vesicular structures are recognizable, which likely correspond to the club-shaped rhoptries, and are bundled together and tethered at the cell apex. Beneath this are two other internal structures that may represent the apicoplast and/or lobes of the single mitochondrion of the parasite. Finally, near the posterior end of the parasite is a large oval structure corresponding to the cell nucleus. In total, five boundaries are assigned with characteristics described in detail in Table S1: including the cell membrane or boundary, an apical bundle resembling the rhoptries where tubules converge near the conoid (Sohn *et al.*, 2011 ▸; Speer *et al.*, 1999 ▸), distinct but possibly interconnected networks of tubules that are more centrally located (mitochondria and/or possibly the apicoplast), and a nuclear region (Fig. 5 ▸ and Table S1). The volumes of these segmented regions range from 1.7% to 13.1% of the total cell volume (Table S1). The structures sit in close proximity to each other within the cell as can be appreciated from projection views of the traced out regions viewed from all three primary axes and a series of projections at various angles along one of the primary axes (Fig. 5 ▸ and Video S1).

### 4.2. Discussion   

We have developed a three-dimensional cryo-CDI method and determined the three-dimensional structure of a whole cell in a frozen-hydrated state. The reconstructed three-dimensional density of the isolated tachyzoite reveals its characteristic banana shape with a three-dimensional resolution of ∼74–99 nm. The distribution of the major organelles in the tachyzoite is polarized, with more dense structures packed near the apical end of the cell. From these structures we have identified the tachyzoite nuclear region, several dense networks of tubules resembling the rhoptries, and a conoid-like structure from which these tubules emanate at the apical tip of the cell. The size and respective structures observed in the tachyzoite are in agreement with previously published negative-stain thin-section transmission electron microscopy (TEM) studies of *N. caninum* and *T. gondii* tachyzoites (Speer *et al.*, 1999 ▸). These TEM studies reported that micronemes, rhoptries and clusters of dense granules are present in *N. caninum* cells (Speer *et al.*, 1999 ▸). Although we cannot definitively assign each of these structures in our three-dimensional reconstruction, the sizes of structures we observed are consistent with those delineated from electron micrographs. The shape of our cell is distinctive of *N. caninum* tachyzoites, as observed in thin-section TEM images (Speer *et al.*, 1999 ▸). The structure of the tachyzoite, as well as those observed by others (Speer *et al.*, 1999 ▸; Schatten & Ris, 2002 ▸), shows a persistently unchanging outer membrane, whose bound is smooth and shows no ruffles. The most discernable sub-cellular compartments are a bundle of club-shaped rhoptries emanating from the conoid, a basket-shaped structure composed of microtubules at the apical end of the cell, and the nucleus, which sits mid-posterior within the parasite (Sohn *et al.*, 2011 ▸; Speer *et al.*, 1999 ▸). Although the tachyzoite we observed is not perfectly round in its shortest dimension due to the geometric constraints imposed by the substrate, it retains its expected characteristic three-dimensional shape.

In order to improve the three-dimensional resolution of cryo-CDI to the ∼10 nm level, further instrument development is needed. First, the ESRF is undergoing a major source upgrade and the coherent X-ray flux of beamline ID10 is expected to increase by a factor of 20 (the ESRF upgrade is detailed at http://www.esrf.eu/about/upgrade). Second, most of our current data acquisition time (∼5 h per tilt series) has been used to manually align the sample to the X-ray beam at each tilt angle. Development of an automated sample mounting and alignment system for three-dimensional cryo-CDI, similar to what has been widely implemented in protein crystallography (Snell *et al.*, 2004 ▸), will substantially reduce our data acquisition time. Third, our current three-dimensional cryo-CDI instrument utilizes a 100 K nitrogen gas stream to preserve the specimen during data acquisition and the whole system is exposed in air. Keeping the cryo-CDI instrument in a helium chamber and using a liquid-nitrogen-cooled helium gas stream will reduce the ice buildup during the data acquisition and allow us to increase the exposure time per tilt angle. Fourth, our current experiment utilizes a MAXIPIX detector with four modules and an array size of 516 × 516 pixels. In order to achieve higher resolution of whole frozen-hydrated cells, a MAXIPIX detector with more modules will be needed; these and other such detectors exist and are under rapid development. Finally, while CDI can in principle achieve higher spatial resolution and have higher photon efficiency than zone-plate-based X-ray microscopy, the latter can directly acquire images and the three-dimensional tomographic reconstruction of cells is fast (Sakdinawat & Attwood, 2010 ▸; Weiß *et al.*, 2000 ▸; Le Gros *et al.*, 2005 ▸; Schneider *et al.*, 2010 ▸; Meirer *et al.*, 2011 ▸). These two forms of microscopy may be combined in a single instrument to make quantitative three-dimensional X-ray imaging of biological specimens even more powerful. These improvements will surely reduce the data acquisition time and improve the data quality, resulting in more robust and higher-resolution three-dimensional structures of cells in their native state.

## 5. Conclusion   

In conclusion, we demonstrate cryo-CDI in three dimensions for the first time by imaging a whole frozen-hydrated *N. caninum* tachyzoite at a three-dimensional resolution of ∼74–99 nm. Our three-dimensional reconstruction reveals the three-dimensional morphology and sub-cellular organization of this large and uniquely structured parasitic pathogen. This structure represents an important experimental milestone toward high-resolution lens-less cryo-imaging of biological specimens in three dimensions. The resolution of this technique is ultimately limited by radiation damage to the samples. We now, in three dimensions, and others previously in two dimensions (Huang *et al.*, 2009 ▸; Lima *et al.*, 2009 ▸), have shown that maintaining samples in a frozen-hydrated state can substantially mitigate radiation damage. Collectively, cryo-CDI efforts to date demonstrate a concerted effort toward realizing the three-dimensional imaging of whole cells at ∼10 nm resolution (Howells *et al.*, 2009 ▸; Shen *et al.*, 2004 ▸). The realization of this aim will open an important venue for revealing the three-dimensional cellular architecture of whole cells in their natural state. Lastly, as new, brighter and highly coherent X-ray sources continue to emerge worldwide (Popmintchev *et al.*, 2012 ▸; Miao *et al.*, 2015 ▸), our work presents a vision of what the field may ultimately achieve: the routine collection of high-resolution quantitative three-dimensional structural information from cells in their native state. These studies are important building blocks for the better structural understanding of cells, and may lead to advances in the fields of imaging, biology and medicine, as well as the general realm of biomaterials.

## Supplementary Material

Table S1 and Figs S1-S4. DOI: 10.1107/S205225251501235X/it5007sup1.pdf

Video S1: 3D isosurface rendering of the model of the reconstructed frozen-hydrated N. caninum tachyzoite with the five colored regions detailed in Table 2. . DOI: 10.1107/S205225251501235X/it5007sup2.mov

## Figures and Tables

**Table 1:** Summary of sample and experiment parameters, data statistics and results

Experiment design and data analysis	
Sample and environment	
Sample (strain)	*N.caninum* tachyzoite (NC1)
Host cell	Human foreskin fibroblasts
Preparation method	Extrusion and normal egress
Ambiance	
Support	Si_3_N_4_ membrane (50nm thick)
Temperature	100K (cryostream)
Local environment	Thin liquid film
Buffer composition	PBS, 10% glycerol, 7% trehalose

Instrumentation and experiment design	
Workstation	ID10 beamline, ESRF
Beam parameters	
Energy (wavelength)	8keV (1.55)
Beam slit size	7m 7m
Distance to sample	0.5m
Intensity at the sample position	4.99 10^7^photonss^1^

Data collection	
Number of projections	72
Exposure time per projection	100s
Sample-to-detector distance	5.29m
Linear oversampling ratio	O_*x*_ = 3.3, O_*y*_ = 6.0, O_*z*_ = 8.1
Tilt range	60.6 to +50.9
Total radiation dose	4.55 10^8^Gy

Phasing method and structure rendering	
Primary processing and analysis	Custom MatLab script
Phase retrieval algorithm (data type)	OSS (assembled 3D matrix)
Number of iterations (independent seeds)	1000 (100)
Segmentation (rendering software)	Manual, threshold-based (*Amira*, *3dmod*, *Chimera*)
