# Supplementary material for: Three-dimensional coherent X-ray diffractive imaging of whole frozen-hydrated cells
Source: IUCrJ. 2015 Aug 20;2(Pt 5):575–83. doi: 10.1107/S205225251501235X (PMC4547825; doi:10.1107/S205225251501235X)
Supplement: Supplementary file 1 [file m-02-00575-sup1.pdf]

IUCrJ

Volume 2 (2015)

Supporting information for article:

Three-dimensional coherent X-ray diffractive imaging of whole, frozen-hydrated cells

Supporting information

Table S1 Summary of the five regions comprising the isosurface model of the reconstructed 3D tachyzoite. Regions are numbered according to their presentation in Fig. 5.

	**Region**	**Color**	**Area (μm^2)**	**Volume (μm^3)**	**% of Cell Volume**
1	Cell Boundary	Pearl	20.4	5.7	100
2	Rhoptries	Red	4.8	0.4	6.5
3	Possible Apicoplast	Orange	3.1	0.3	4.9
4	Nucleus	Brown	8.6	0.8	13.1
5	Mitochondrion	Blue	1.9	0.1	1.7

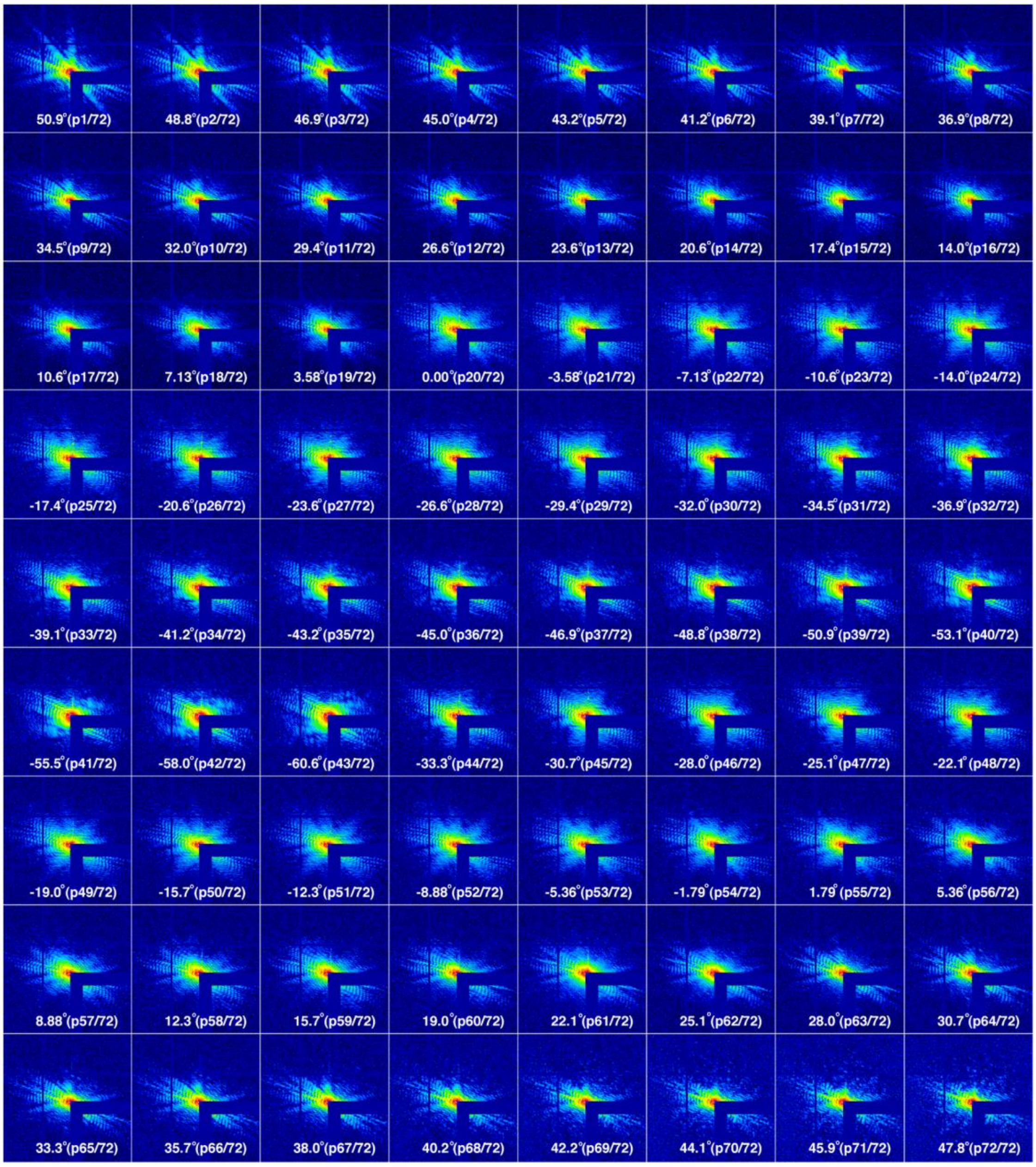

Figure S1 A representative tilt series of 72 coherent X-ray diffraction patterns measured from a frozen-hydarted *N. caninum* tachyzoite. The diffraction patterns are numbered by the tilt angles (ranging from -60.6° to 50.9°) and corresponding projections, in the order in which they were acquired. The exposure time of each pattern is 100s and a beam stop shadow is visible in all patterns (two bars in the low right quadrant). The diffraction patterns are shown in a logarithmic scale.

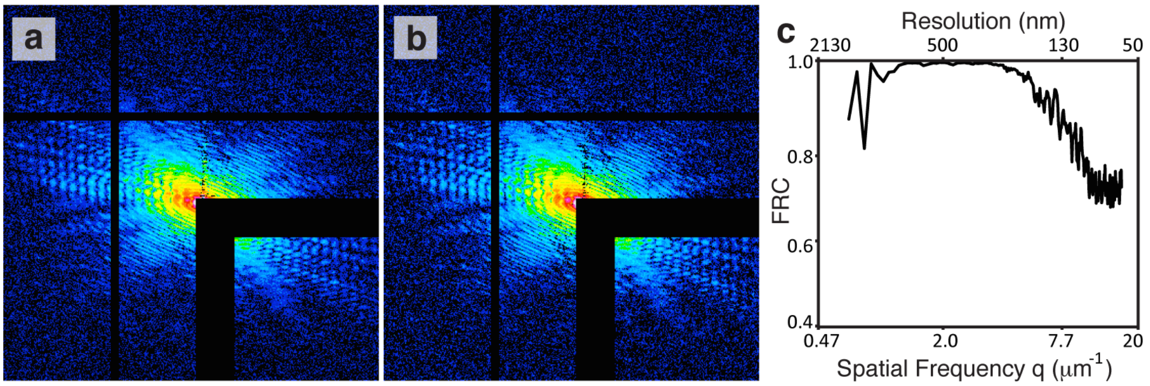

Figure S2 Comparison of two independently measured diffraction patterns at 0° at differernt acquisiton time. (**a, b**) Two independent diffraction patterns of the same tilt angle (0°) collected from the frozen-hydarted *N. caninum* tachyzoite during the acquistion of the tilt series. (**c**) Fouir ring corrleation (FRC) comparision between the two 0° diffraction patterns (**a, b**), showing no appreciable difference in the diffraction patterns. The small dip at the low spatial frequency is due to the beamstop at the center.

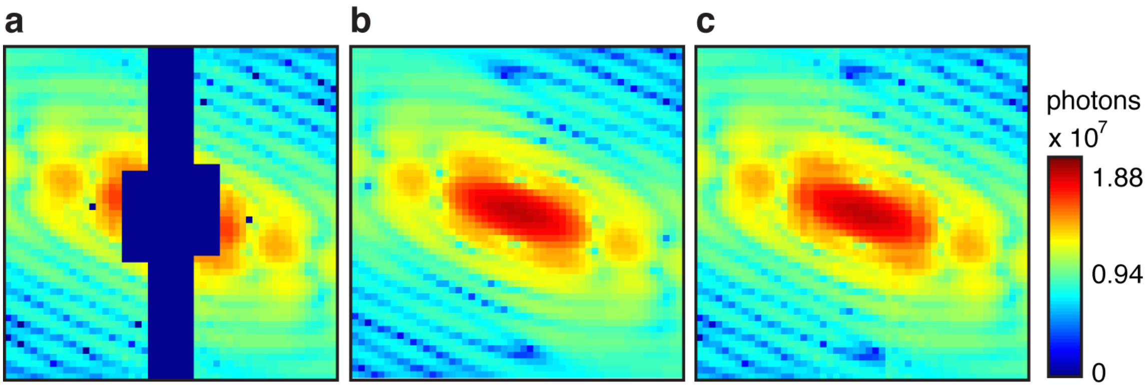

Figure S3 Retrieval of the central speckle intensities from the 3D reconstruction of the *N. caninum* cell. **(a)** A central slice through the assembled 3D diffraction pattern obtained from the measured tilt series. The missing center size of the diffraction pattern (blue region) is comparable to that of the centro-speckle, which is important for high quality image reconstruction. **(b)** The corresponding slice through the 3D diffraction intensities calculated from the 3D recosntructed cell. **(c)** Same as (**a**) with the missing intensities obtained from **(b),** indicating a high consistency between the measured and retrieved central speckle intensities.

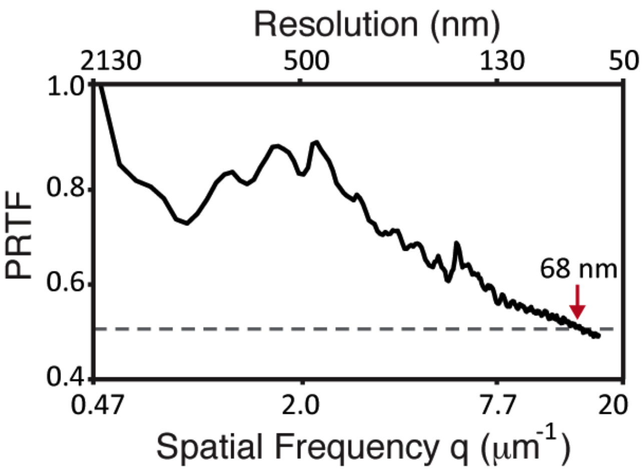

Figure S4 The phase retrieval transfer function (PRTF) calculated from the 3D reconstructions of the frozen-hydarted *N. caninum* cell. According to the PRTF=0.5 criterion, a resolution of approximately 68 nm was estimated.

Video S1 3D isosurface rendering of the model of the reconstructed frozen-hydrated *N. caninum* tachyzoite with the five colored regions detailed in Table 2.
